# Study on the Construction Mechanism and Survival Strategy of Important Estuarine Zooplankton Communities in Qinhuangdao Sea, Bohai Sea, China

**DOI:** 10.3390/biology14121675

**Published:** 2025-11-25

**Authors:** Long Yun, Xiangping Xue, Zhaohui Sun, Xinjing Xu, Jiangwei Zan, Gao Meng, Xinye Zhao, Gao Song, Fei Si, Yong Song

**Affiliations:** 1 College of Life Sciences and Technology, Tarim Research Center of Rare Fishes, State Key Laboratory Incubation Base for Conservation and Utilization of Bio-Resource in Tarim Basin, Tarim University, Alar 843300, China; 10757232147@stumail.taru.edu.cn; 2 Hebei Key Laboratory of the Bohai Sea Fish Germplasm Resources Conservation and Utilization, Beidaihe Central Experiment Station, Chinese Academy of Fishery Sciences, Qinhuangdao 066100, China; xuexp@bces.ac.cn (X.X.); sunzh@bces.ac.cn (Z.S.); xuxj@bces.ac.cn (X.X.); zanjw@bces.ac.cn (J.Z.); mengg@bces.ac.cn (G.M.); zhaoxy@bces.ac.cn (X.Z.); gaos@bces.ac.cn (G.S.); 3 Bohai Sea Fishery Research Center, Chinese Academy of Fishery Sciences, Qinhuangdao 066100, China

**Keywords:** estuarine area, zooplankton functional groups, niche, interspecific association

## Abstract

The objectives of this study were to investigate the zooplankton community in the important estuarine areas of Qinhuangdao Sea in the Bohai Sea, analyze the variation characteristics of the zooplankton community, and explore the construction and evolutionary mechanisms of its community structure and function. We conducted zooplankton monitoring in the four seasons of 2024 at Daihe Estuary (DHK), Yanghe Estuary (YHK), Shihe Estuary (SHK), Tanghe Estuary (THK), and Luanhe Estuary (LHK). The results showed that the diversity index and richness index in summer were significantly higher than those in winter (*p* < 0.05). Based on the body size, feeding habits, reproduction types, life cycles, and escape abilities of zooplankton, the zooplankton in the ecosystem were divided into 17 zooplankton functional groups. The zooplankton examined in this study were divided into 11 functional groups. Except for THK, the dominant functional groups in other estuaries in all seasons were SCF (Small copepods and claocera filter feeders) and LCF (Large copepods and claocera filter feeders); PP (Protozoan photosynthetic autotrophs) and PB (Protozoan bacterivores) functional groups dominated THK. Pearson correlation analysis and RDA multivariate statistical analysis revealed that interspecific competition, PO_4_^3−^-P, NO_2_^−^-N, and SAL were the primary driving factors influencing community construction. The spatial-temporal variations in T, PO_4_^3−^-P, NO_2_^−^-N, and SAL in the five estuaries were evident, and the pH and DO in the water environment exhibited a high degree of overlap in seasonal changes. The density of zooplankton functional groups was significantly positively correlated with PO_4_^3−^-P, NO_3_^−^-N, NO_2_^−^-N, NH_4_^+^-N and SAL (*p* < 0.05). Furthermore, the SCF and LCF functional groups played a dominant role. According to the analysis of niche overlap and AC interspecific association, it was shown that zooplankton populations in small-scale areas mostly exhibited negative correlations dominated by competition and predation. In contrast, the functional groups of zooplankton in large-scale areas mostly demonstrated mutually synergistic facilitative relationships. The results of this study showed that interspecific relationships are more important for the community structure of zooplankton. This study contributes to understanding the responses of primary producers and primary consumers to water environmental changes in estuarine ecosystems, laying a foundation for further analyzing the mechanisms of zooplankton community construction and survival strategy selection in special estuarine habitats. It also provides references for the scientific management of estuarine areas and the avoidance of pollution risks.

## Simple Summary

This study investigated the zooplankton community in five estuaries of the Qinhuangdao Sea, Bohai Sea, through seasonal monitoring in 2024. A total of 69 zooplankton species were identified and classified into 11 functional groups. The results revealed significant spatiotemporal variations, with higher biodiversity in summer than in winter. The community structure at Tanghe Estuary was distinctly different from the others. Our findings indicate that interspecific competition, PO_4_^3−^-P, NO_2_^−^-N, and SAL were the key drivers shaping the community. Furthermore, the Random Forest model identified interactions among MCF (Middle copepods and claocera filter feeders), SCF (Small copepods and claocera filter feeders), and LCF (Large copepods and claocera filter feeders) functional groups as the most important positive factors influencing the functional structure of the community.

## 1. Introduction

The Bohai Sea in China is a semi-enclosed inland sea, bounded by the line connecting Laotieshan Cape of the Liaodong Peninsula via the Miaodao Islands to Penglai Cape at the northern end of the Shandong Peninsula in the east, separating it from the Yellow Sea. It is a semi-enclosed inland sea surrounded by land. The Bohai Sea is situated in the temperate zone, falling within the warm-temperate semi-humid continental monsoon climate zone. It is characterized by distinct seasonal variations: dry with little rainfall in spring, humid and rainy in summer, clear and dry in autumn, and cold with little snow in winter [1]. Statistics show that there are up to 40 rivers along the Bohai Rim, among which there are representative rivers in the Qinhuangdao Sea area of the Bohai Sea, such as the Luanhe River, Daihe River, Yanghe River, Datanghe River, Shihe River, etc. The sea area near Qinhuangdao belongs to the regular diurnal tide zone. In contrast, the area south of the Luanhe Estuary is an irregular diurnal tide zone, exhibiting a trend of low tides in winter and high tides in summer. Due to the influence of tidal action and special climatic characteristics, the hydrological characteristics of the estuarine area exhibit obvious seasonal variations. In summer, with abundant rainfall, incoming rivers carry large amounts of sediment and organic matter, often accumulating to form various types of wetlands and special estuarine hydrological features in the estuaries. This makes the estuarine water quality fertile and rich in forage organisms, serving as habitats for the survival, reproduction, and feeding of numerous fishery organisms [2].

Zooplankton widely exist in various types of water bodies and are important components in maintaining the structure and function of aquatic ecosystems. Being extremely sensitive to environmental changes, zooplankton can serve as biological indicators of different environmental conditions [3]. Environmental factors can alter the relationships within zooplankton communities and are crucial determinants of planktonic community structures. Planktonic communities also influence each other, giving rise to interactions of mutual antagonism or collaboration among different groups [4,5]. A functional group refers to a collective of species with similar structural or functional characteristics, representing a classification of species based on their analogous traits or behaviors within an ecosystem. Studies on functional groups can more directly reflect the ecological processes through which the environment impacts biological communities. Plankton are classified based on their size, feeding habits, reproductive methods, life cycle, and ability to evade predators. Therefore, the construction of zooplankton functional groups helps elucidate the relationship between zooplankton communities and ecosystems, providing insights into the mechanisms of zooplankton community assembly [6,7]. Through the study of functional groups, the ecological processes by which the ecological environment affects biological communities can be more directly reflected. This helps to elaborate on the relationship between zooplankton communities and ecosystems, providing a reference for the mechanisms of zooplankton community construction [6,8]. By examining the diversity and biomass changes in plankton, as well as the distribution patterns of zooplankton and their interspecific and intraspecific relationships, variations in fish productivity across different locations in this region can be inferred. This provides a basis for better protection and management of the area. In Chai Z’s research, the mutual influences among plankton communities were also mentioned. Zooplankton communities can influence one another, with interactions between different communities exhibiting either mutual antagonism or cooperation. Plankton communities can be classified using various methods based on their vital characteristics and trophic traits [9]. For example, in Zhao Yuxi’s study on the distribution patterns and interspecific relationships of zooplankton in reservoirs during different periods, it was found that interspecific interactions had a greater influence on species composition and distribution than external environmental factors [10].

Ruizhi and colleagues conducted a study on the taxonomic and functional group composition of protists in high-altitude wetlands, proposing that protists resist the extreme environmental conditions of alpine wetlands through close cooperation, with their distribution driven by environmental selection. This research, carried out in the Medika Wetland (the highest wetland of international importance), revealed that protist communities form co-occurrence networks dominated by positive interactions, enabling them to collectively adapt to harsh environments, such as low temperatures and variable salinity [11]. Only a few basic studies have been conducted, including the following: Based on the survey data from four voyages conducted in 2013–2014, it was analyzed that a total of 43 zooplankton species were identified in the Yellow River Estuary and its adjacent waters, with the community structure showing obvious seasonal variations [12]. Through sampling surveys during the dry season, normal flow season, and wet season in 2015, it was found that there were 30 zooplankton species belonging to 6 categories in the oyster-spawning ground of the Yellow River Estuary and its adjacent waters, with significant spatial and seasonal differences in their abundance. The zooplankton community structure was optimal in the dry season, and the environmental quality of the surveyed waters was evaluated as moderately polluted [13]. Current studies in the literature mainly focus on water bodies with stable hydrology, while research on estuaries—one of the most productive ecosystems on Earth—remains scarce. Frequent water exchange in these regions creates special habitats. Analyzing the interspecific relationships of zooplankton functional groups and their primary driving factors in estuarine areas is of great significance for accurately identifying pollution status; the community stability was analyzed through the interspecific association analysis of ecological niche AC, understanding the responses of primary producers and primary consumers to environmental changes in estuarine ecosystems, and providing references for scientific management and pollution risk avoidance in estuaries.

This study conducts four-season tracking monitoring of five key estuaries in Qinhuangdao, Bohai Sea, to analyze the community assembly of zooplankton in the five estuarine areas and the spatiotemporal variation patterns concerning water environmental factors. The research aims to (1) identify the main driving factors influencing zooplankton community assembly; (2) clarify inter-community relationships and construct co-occurrence networks; and (3) integrate niche overlap rates, interspecific associations, and zooplankton functional groups to explore the niche-linkage relationships of zooplankton functional groups. This study aims to provide new insights for further interpreting the ecological patterns and community assembly mechanisms of zooplankton in estuarine ecosystems, as well as for assessing water ecological health.

## 2. Materials and Methods

### 2.1. Study Area and Station Setting

**Figure 1:**
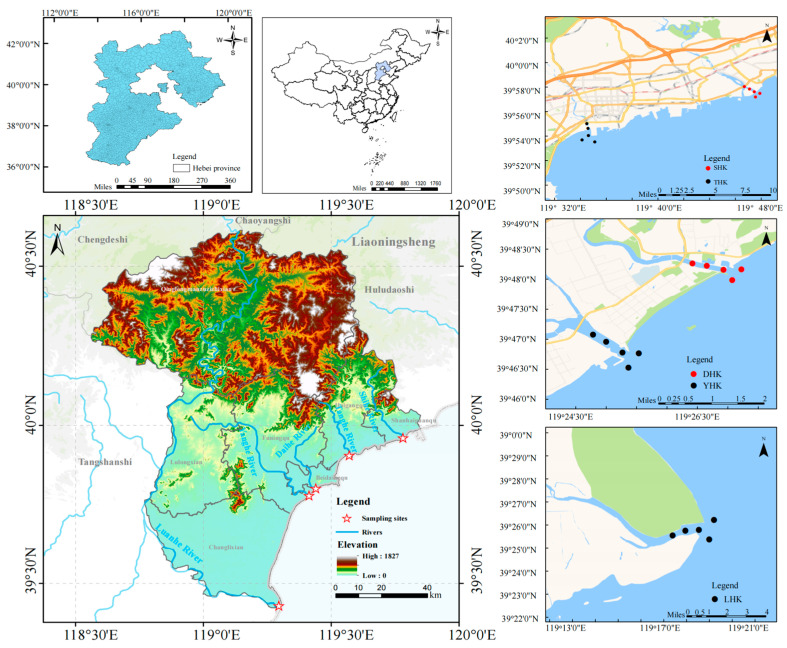
Sampling of five estuaries in Qinhuangdao city.

This study focuses on the coastal zone of Qinhuangdao in the Bohai Sea, with a total coastline length of 144.84 kilometers located in the central Bohai Sea [1]. Multiple rivers flow into the Bohai Sea in the Qinhuangdao area. The monitored estuarine rivers, from north to south, are the Shihe River, Tanghe River, Daihe River, Yanghe River, and the Luanhe River, the largest river in the southern part. According to the characteristics of the field water area, sampling points are arranged longitudinally along the estuary, covering freshwater, brackish water, and saltwater zones; the stations were set up as shown in Figure 1. Surveys were conducted at five estuaries, including Shihe Estuary, Tanghe Estuary, Daihe Estuary, Yanghe Estuary, and Luanhe Estuary, in March, June, September, and December 2024.

### 2.2. Sample Collection and Determination Method

#### 2.2.1. Physicochemical Indexes of Water Bodies

Portable pH/conductivity/dissolved oxygen meter (Shanghai Sanxin, SX836) and salinity meter (Ohaus, ST20S) were used to measure the on-site water environment index. The on-site measurement data of the monitoring station include water temperature (WT), pH, dissolved oxygen (DO) and salinity (SAL). The determination of other water quality indicators was carried out in the laboratory with reference to standards such as (Methods for Monitoring Water and Wastewater). The four water quality indicators include ammonia nitrogen (NH_4_^+^-N), nitrate nitrogen (NO_3_^−^-N), nitrite nitrogen (NO_2_^−^-N), and dissolved phosphate (PO_4_^3−^-P) [14]. Nitrite was determined by the naphthylethylenediamine spectrophotometric method; ammonia nitrogen by the indophenol blue spectrophotometric method; and reactive phosphate by the phosphomolybdenum blue spectrophotometric method [15,16,17].

#### 2.2.2. Plankton

According to Specifications for Marine Monitoring—Part 7: Offshore Pollution Ecological Survey and Biological Monitoring (GB 17378.7-2007) [18], zooplankton samples are collected by vertical towing from the seabed to the surface using Type I Shallow-Water Plankton Net (hereafter referred to as Type I Net) and Type II Shallow-Water Plankton Net (hereafter referred to as Type II Net). The collected samples are fixed and preserved by adding formaldehyde solution at 5% of the sample volume. Zooplankton samples are analyzed via the individual counting method for identification and enumeration, and the zooplankton biomass refers to the wet weight biomass based on samples collected by the Type I Net. The methods for plankton sampling and analysis were conducted in accordance with Specifications for Marine Surveys—Part 6: Marine Biological Surveys (GB/T 12763.6-2007) [19]. The classification and identification of plankton were carried out with reference to Atlas of Marine Plankton in China [20], Fauna Sinica—Arthropoda: Crustacea: Freshwater Copepoda, Fauna Sinica—Arthropoda: Crustacea: Freshwater Cladocera, Monograph of Freshwater Rotifera in China [21,22].

The abundance of zooplankton is calculated according to the following formula:

(1) $$N=\frac{n}{v}$$

where *N* is the zooplankton abundance (ind.m^−3^); *v* is the volume of filtered seawater (m^3^); and *n* is the number of zooplankton individuals (ind.). The zooplankton biomass refers to the wet weight biomass based on samples collected by the Type I Net [23].

Based on the body size, feeding habits, reproduction types, life cycles, and escape abilities of zooplankton, the zooplankton in the ecosystem were divided into 17 zooplankton functional groups (Table 1) [24]. A total of 57 species, including *Cladocera*, *Copepoda*, *Rotifera*, *Protozoa*, and *Noctiluca* (other species were all larvae and excluded from functional group analysis), were divided into 11 functional groups.

### 2.3. Data Processing and Analysis

#### 2.3.1. Calculation of Dominant Species

The calculation formula for the dominant species of zooplankton is as follows [25]:

$$Y=\left(\frac{n_{i}}{N}\right)f_{i}$$

In the formula, the variables are as follows:

*n_i_*: the proportion of individuals belonging to the *i*-th species relative to the overall number of individuals;
*N*: the total number;
*f_i_*: The frequency of occurrence of the *i*-th species. When *Y* > 0.02, this species is regarded as a dominant species.

#### 2.3.2. Biodiversity

Diversity index of zooplankton (Shannon–Wiener, *H*′).

Shannon–Wiener diversity index (*H*′) calculation formula:

$$H’=-\sum p_{i}\times {log}_{2}p_{i}$$

Richness index of zooplankton (Margalef, *d*).

Margalef richness index (*d*) calculation formula:

$$d=(S-1)/{log}_{2}N$$

Evenness index of zooplankton (Pielou, *J*′).

Pielou evenness index (*J*′) calculation formula:

$$J’=H’/{log}_{2}S$$

Zooplankton diversity index (Simpson, *D*).

Simpson diversity index (*D*) calculation formula:

$$D=1-\sum {Pi}^{2}$$

*S*: the number of species in the community;
*N*: the total number of individuals observed in the quadrat;
*Pi*: the proportion of the number of individuals of the *i*-th species at this station out of the total number of individuals.

In the above analysis, PRIMER 5.2.8 was used to calculate the Margalef species richness index (*d*), the Shannon–Wiener diversity index (*H*′), the Simpson dominance index (*D*), and the Pielou evenness index (*J*′) to measure and analyze the biodiversity of the zooplankton community [26].

#### 2.3.3. Redundancy Analysis (RDA)

DCA analysis was carried out using Canoco for Windows 4.5 software, and the SD value was >3. Therefore, RDA was carried out on species data and related environmental factor data to investigate the effects of various environmental factors on zooplankton [27].

#### 2.3.4. NMDS + PERMANOVA Analysis

The NMDS algorithm was used to study the similarity or difference of the composition of Phytoplankton functional (the data adopts the biomass of zooplankton functional groups, with each species assigned to a separate functional group) group communities, and PERMANOVA (Adonis) was used for testing and identification. Nonparametric multivariate analysis of variance was used for sample distance (Bray–Curtis). To reveal the distribution differences of Zooplankton functional groups among different sampling sites, the NMDS (Non-metric Multidimensional Scaling) algorithm was employed for dimensionality reduction analysis of community compositions to visualize the similarities between samples. The significance of inter-group differences was tested using PERMANOVA with 999 permutations. For evaluating the stability of stress values, the reliability of results was verified through 200 repeated calculations [28].

#### 2.3.5. Prediction of New Random Forest Model

Corresponding analyses were conducted using the Random Forest extension package (rfPermute) under R version 3.1. The R value was computed via the tidyverse package, while plot exploration was carried out with the ggplot2 package in R. Errors were quantified using out-of-bag (OOB) data—this dataset was further used to determine the relative importance of each functional group involved in the analysis, and these groups were subsequently ranked based on the resulting importance values. The random forest model employed 1000 decision trees, with performance evaluated using 5-fold cross-validation (where the training set accounted for 80% and the validation set for 20% of the data). The feature splitting parameter mtry was set to the square root of the total number of features to ensure the reproducibility and scientific rigor of the results [29,30].

#### 2.3.6. Correlation Analysis and Mantel Test Correlation Test

After the Zooplankton functional group communities were processed by log10 to make them more normal, the average clustering method and Euclidean distance algorithm were used to conduct Pearson correlation analysis to explore the correlation between different Phytoplankton functional groups communities and environmental factors [31]. One-way ANOVA was used to analyze environmental factors with IBM SPSS Statistics 21 software to study the differences of environmental factors at different times, and *p* < 0.05 was used to indicate significant differences. In order to test the significance of environmental factors and Zooplankton functional groups communities, Mantel test correlation was used, and Bonferroni method was used for p value correction. The analysis result of Mantel test heat-map plot was generated using the R 4.3.1 software package “vegan”. The Variance Inflation Factor (VIF) was calculated to ensure that there is no collinearity among environmental variables. All the above analyses were carried out with the Homogeneity test and normal distribution correction.

#### 2.3.7. Study on the Model of Niche Width and Interspecific Relationship

Using R version 3.1, the spaa package was used for data analysis and plotting. The calculation formulas are as follows: Niche breadth (Bi) was measured using Levins’ index [32], and Niche overlap (Oik) was calculated with Pianka’s overlap index.

$$Bi=\frac{1}{\sum_{j=1}^{r}\;P_{ij}^{2}}$$

where Bi is the niche breadth of the *i*-th species, j represents the quadrat, and r denotes the number of quadrats.

$$Oik=1-\frac{1}{2}\sum_{j=1}^{r}\left|p_{ij}\right.-\left.p_{kj}\right|$$

where Oik is the niche overlap coefficient between species i and k, and P_ij_ and P_kj_ are the abundances of species i and k at sampling site j, respectively.

Interspecific Association:

The variance ratio method (VR) was used to measure the overall (association), and the χ^2^ test was combined to qualitatively determine interspecific associations [33].

The overall association was calculated using the variance ratio method with the following formulas:

Variance of species relative abundance:

$$\sigma_{T}^{2}=\sum_{i=1}^{a}p_{i}{\left(1-pi\right)}^{2}$$

Variance of species richness:

$$S_{T}^{2}=\frac{1}{N}\sum_{j=1}^{N}{\left(T_{j}-t\right)}^{2}$$

Variance Ratio:

$$VR=\frac{S_{T}^{2}}{\sigma_{T}^{2}}$$

W=VR⋅N

Formula Description: S: Total number of dominant zooplankton species; N: Number of sampling sites; T_j_: Number of dominant zooplankton species in sampling site j. If VR > 1: Overall positive association; if VR < 1: Overall negative association; if VR = 1: No interspecific association. If W < χ^2^_0.95_ (N) or W > χ^2^_0.05_ (N), overall connectivity obvious (*p* < 0.05); if χ^2^_0.95_ (N) < W < χ^2^_0.05_ (N), overall association was not significant (*p* > 0.05).

Interspecific Association Test:

The stability of community structure and interspecific relationships can be reflected by interspecific associations, which play a positive role in the prediction of zooplankton succession [34]. When the number of positive correlations is greater than that of negative correlations, the community tends to be stable; when the number of positive correlations is less than that of negative correlations, the community is in the successional process. In this study, the point correlation coefficient (Φ) was used to test and quantify the association and association strength between each pair of dominant species [35]:

$$pcc=\frac{ad-bc}{\left(a+b)(a+c)(b+d)(c+d\right)}$$

a: Number of sampling sites where both species are present; b: Number of sampling sites where both species are present; c: Number of sampling sites where species A is present but species B is absent; d: Number of sampling sites where neither species is present. The PCC (point correlation coefficient) ranges from [−1, 1]; the PCC (point correlation coefficient) ranges from [−1, 1]; the PCC (point correlation coefficient) ranges from [−1, 1].

## 3. Results and Analysis

### 3.1. Composition and Distribution of Zooplankton Species

**Figure 2:**
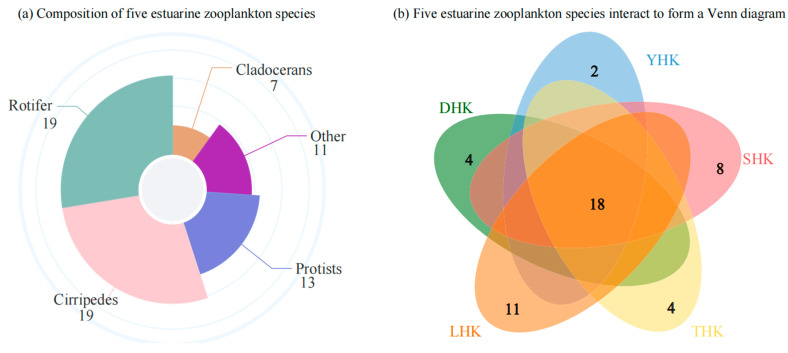
(**a**) Five estuarine zooplankton Species composition. (**b**) The intersection of five estuarine species in a Venn diagram.

The results of zooplankton surveys conducted at 15 sampling sites across five estuaries in Qinhuangdao over four months are shown in Figure 2a. A total of 69 zooplankton species were collected (including 16 Species identified to genus; Species is a biological juvenile), including 19 rotifer species (27.54%): Phylum Rotifera; 19 copepod species (27.54%): Phylum Arthropoda; 13 protozoan species (18.84%): Phylum Protozoa; 11 species from other groups (15.94%): Phylum Arthropoda (Crustacea), Phylum Mollusca, Phylum Chaetognatha, and Phylum Tunicata (Urochordata); and 7 cladoceran species (10.14%): Phylum Arthropoda. A total of 18 species were shared among the five estuaries (Figure 2b). Specifically, Luanhe Estuary (LHK) had 11 unique species, Shihe Estuary (SHK) had 8 unique species, Daihe Estuary (DHK) and Taihe Estuary (THK) each had 4 unique species, and Yanghe Estuary (YHK) had only 2 unique species, indicating significant differences in species composition among the regions.

**Figure 3:**
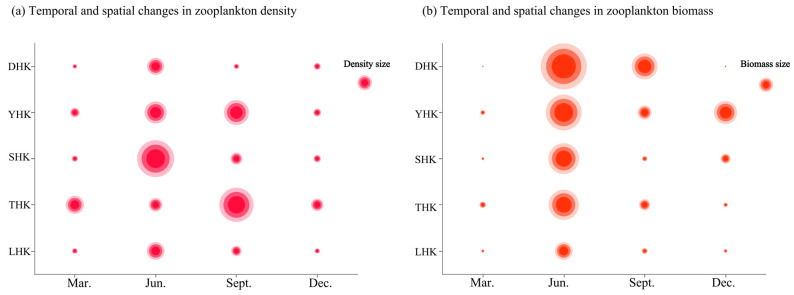
(**a**) Changes in zooplankton density at five estuaries; (**b**) Changes in zooplankton biomass at five estuaries.

The spatiotemporal variation in zooplankton density and consistency is shown in Figure 3a. In different seasons, the species abundance of zooplankton in the five estuaries exhibits relatively obvious fluctuating characteristics. Except for THK and YHK, the other three estuaries showed that the abundance in June was higher than that in other months. The abundance in YHK and THK was the highest in September. Among them, the species abundance in the SHK region in June is higher than that in other months, and the seasonal variation in species abundance is most clearly manifested. Spatial distribution analysis revealed that the species abundance was the highest in SHK. The abundance in THK was close to that in SHK, while the species abundances in DHK and LHK were slightly lower than those in the other three estuaries. The spatiotemporal variation in zooplankton biomass is shown in Figure 3b. Temporal distribution analysis revealed that the biomass in all five estuaries was highest in June, followed by the other three months.

**Figure 4:**
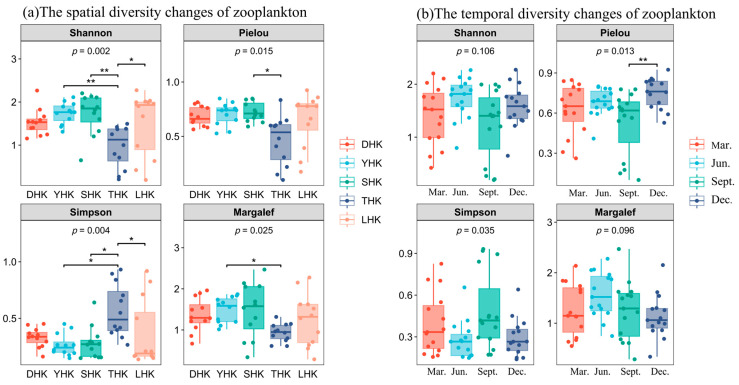
Boxplot of Alpha diversity index for zooplankton. Note: * denotes *p* < 0.05, ** denotes *p* < 0.01.

The spatiotemporal variation in zooplankton diversity in the five estuaries is shown in Figure 4. Spatial analysis of the *Shannon* diversity index among the five estuaries (Figure 4a) revealed significant differences (*p* < 0.01). The diversity index in SHK was higher than that in the other four estuaries, followed by YHK, LHK, and DHK. THK showed a significantly lower diversity index than the others, which was attributed to its lower zooplankton species richness. The Simpson dominance index showed significant differences (*p* < 0.01), with THK being much higher than the other four estuaries. The Pielou evenness index and Margalef richness index showed significant differences (*p* < 0.05), with THK being much lower than the other four estuaries in both metrics. In terms of time scale (Figure 4b), Shannon and Margalef diversity indices showed little difference. The Pielou evenness index showed significant differences among the five estuaries (*p* < 0.05), with extremely significant differences between September and December (*p* < 0.01). The evenness index in September was much lower than that in other months. The Simpson dominance index showed significant differences among the five estuaries (*p* < 0.05). The Simpson advantage index between the five estuaries was significant (*p* < 0.05), with a slight increase in September compared to March and substantially higher than in June and December.

### 3.2. Spatial and Temporal Heterogeneity of Functional Groups of Zooplankton

**Figure 5:**
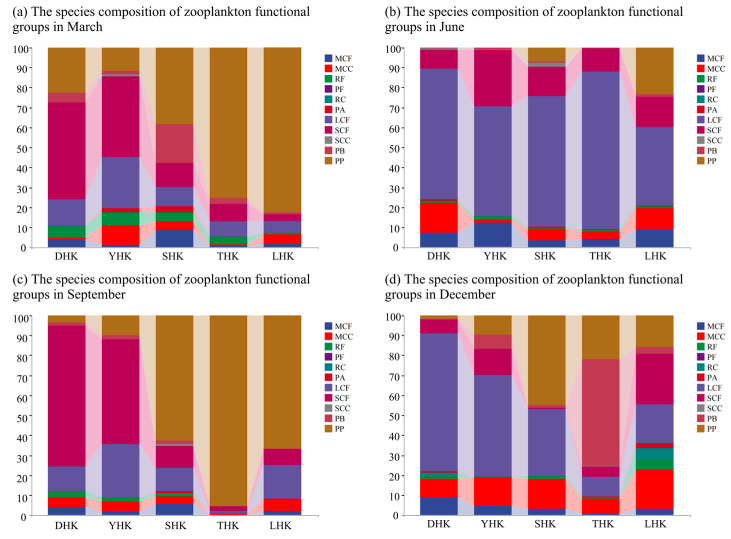
(**a**) Species composition of functional groups in March; (**b**) Species composition of functional groups in June; (**c**) Species composition of functional groups in September; (**d**) Species composition of functional groups in December.

A total of 57 species, including *Cladocera*, *Copepoda*, *Rotifera*, Protozoa, and *Noctiluca* (other species were all larvae and excluded from functional group analysis), were divided into 11 functional groups. The spatial and temporal distribution of functional groups of zooplankton is shown in Figure 5. According to the spatial distribution analysis, the species composition of DHK and YHK in March was dominated by the functional group SCF, accounting for 48.76% and 40.62%, respectively. SHK, THK, and LHK were dominated by the functional group PP, accounting for 38.16%, 75.16%, and 82.57%, respectively. In June, the functional group composition was dominated by the LCF functional group, among which the absolute dominant species, *Calanus sinicus*, played a primary role. Only SHK and LHK found PP functional groups, accounting for 6.81% and 23.25%, respectively. In September, the functional group composition showed significant variations. DHK and YHK were dominated by the SCF functional group, with *nauplii* as the main species. SHK, THK, and LHK were dominated by the PP functional group, with *Noctiluca scintillans* as the key functional species. In THK, *Noctiluca scintillans* underwent a population bloom, contributing 95.37% to the functional group composition. In December, DHK, YHK, and LHK were dominated by the SCF functional group, accounting for 69.1%, 50.99%, and 25.18%, respectively. SHK was dominated by the PP functional group, with *Noctiluca scintillans* as the main species, accounting for 44.73%. THK was dominated by the PB functional group, accounting for 53.53%, with *Tintinnopsis* as the key indicator species of the functional group.

**Figure 6:**
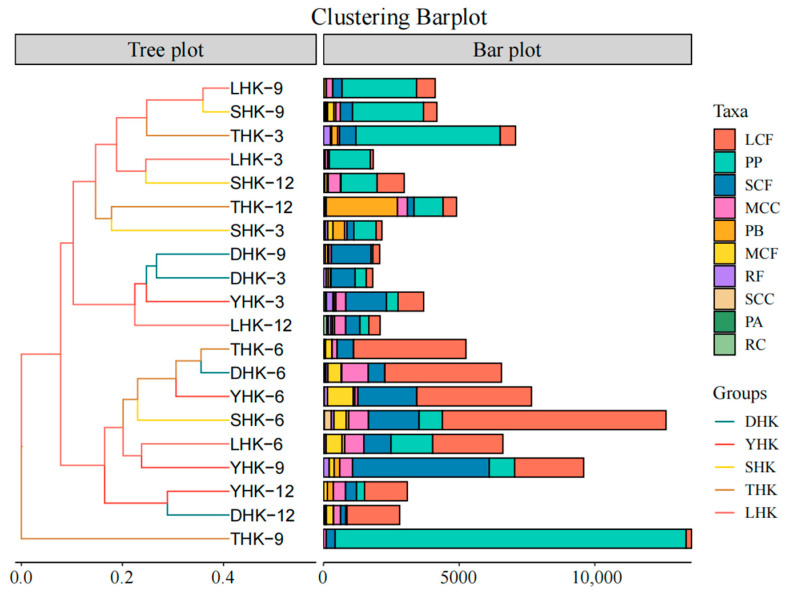
Temporal and spatial variation and similarity analysis of zooplankton functional group composition. Note: The left side shows the similarity analysis, and the right side presents the density composition of zooplankton functional groups.

**Figure 7:**
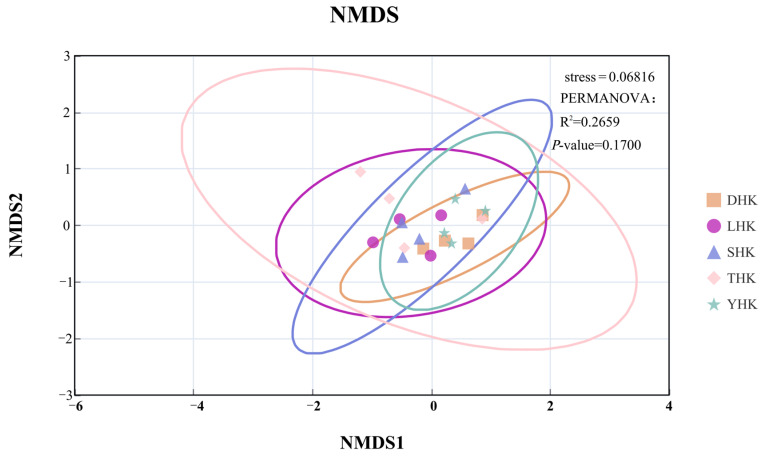
NMDS analysis of functional groups of zooplankton at five estuaries.

The Bray–Curtis similarity coefficient was used to analyze the similarity of functional groups of zooplankton in different estuaries (Figure 6). The five estuarine functional groups are very different. In June, the species composition of DHK and THK was the most similar, indicating the highest similarity. In September, LHK and SHK followed in similarity. The species composition of DHK in March and September was relatively close to that of YHK in March. However, the species composition of THK in September showed significant differences from other estuaries. To explore the clustering relationships of the five estuaries as a whole, this study conducted non-metric multidimensional scaling (NMDS) and cluster analyses. As shown in Figure 7, the overall analysis indicated no significant similarity was observed among the four estuaries (*p* < 0.05).

### 3.3. Temporal and Spatial Characteristics of Physical and Chemical Indexes of Water Bodies

**Figure 8:**
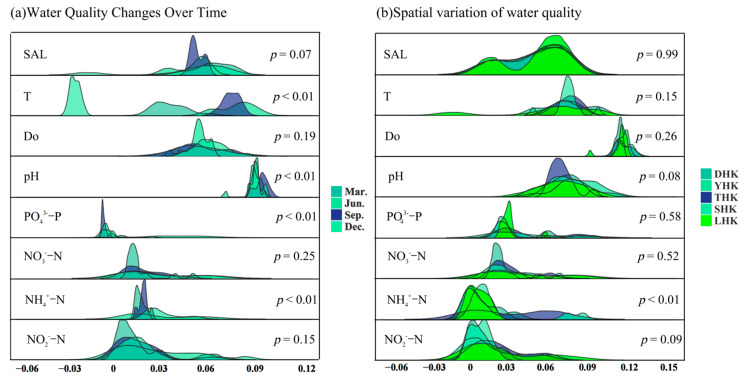
Analysis of the spatial–temporal differences in physical and chemical indexes of water bodies. Note: (**a**) shows temporal heterogeneity; (**b**) shows spatial heterogeneity.

Temporal analysis shown in Figure 8a reveals that pH in June was slightly lower than that in the other three months; PO_4_^3−^-P in December was much higher than in other months; NH4^+^-N nitrogen was higher in June and December, lower in April and September; T was highest in June and lowest in December; both NO_2_^−^-N and NO_3_^−^-N were higher in June and December; and salinity in December was much lower than in the other three months. Except for T, PO_4_^3−^-P and NO_3_^−^-N, other physical and chemical indexes of water bodies showed no significant changes. Temporal analysis of the five estuaries showed PO_4_^3−^-P exhibited significant temporal variations (*p* < 0.01), with the PO_4_^3−^-P index in June significantly higher than that in the other three months; T showed extremely significant variations (*p* < 0.01), with June significantly warmer than other months; NH_4_^+^-N showed significant variations (*p* < 0.01), with June and December significantly higher than April and September.

The spatial variation in water environmental factors is shown in Figure 8b. The T and DO heights of the five estuaries overlap. In terms of environmental factors, NH_4_^+^-N was higher in THK and SHK; pH in LHK was much lower than that in the other four estuaries; NO_2_^−^-N and NO_3_^−^-N were both higher in THK and LHK; Salinity in LHK was much lower than that in the other four estuaries. From a spatial perspective, analysis of the five estuaries showed no significant changes in environmental factors except for NH_4_^+^-N. However, significant spatial variations in NH_4_^+^-N were observed among the five estuaries (*p* < 0.01). The NH_4_^+^-N index in LHK was significantly lower than that in other estuaries, while SHK had the highest PO_4_^3−^-P index.

### 3.4. Analysis of the Interactions Among Zooplankton Functional Groups and Their Correlation with the Aquatic Environment

**Figure 9:**
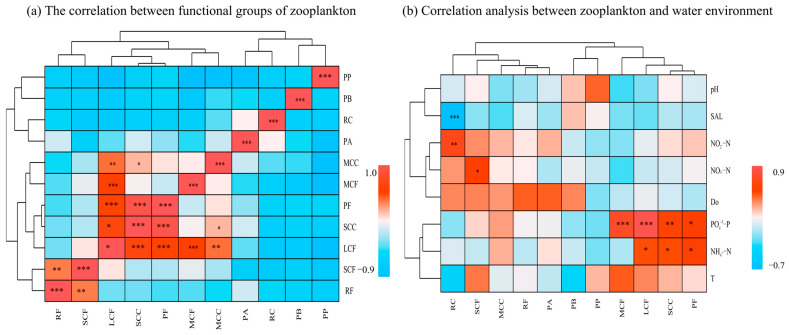
(**a**) Correlation between zooplankton functional groups; (**b**) Correlation analysis between zooplankton functional groups and water environment. Note: * denotes *p* < 0.05, ** denotes *p* < 0.01, *** denotes *p* < 0.001, and no asterisk indicates *p* > 0.05.

**Figure 10:**
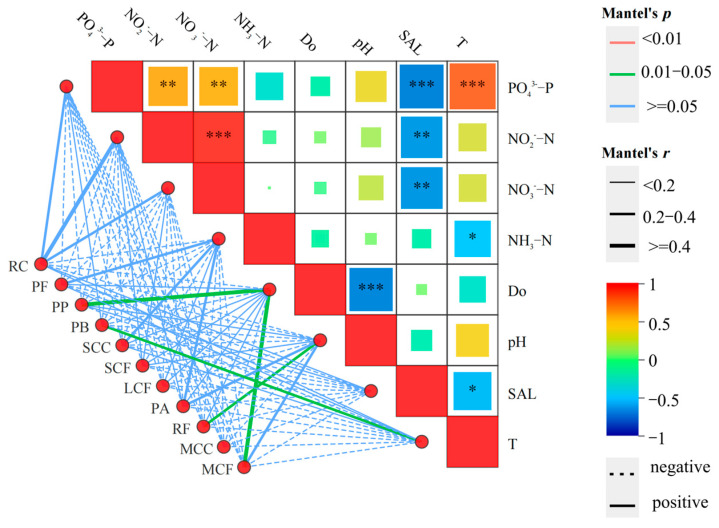
Mantel test heat map of zooplankton functional groups and environmental factors. Note: The width of the line indicates the magnitude of the absolute correlation (Mantel’s *r*), the color of the line shows the range of the significance *p*-value (Mantel’s *p*), and the type of line (solid or dashed) indicates the sign of the correlation coefficient. * denotes *p* < 0.05, ** denotes *p* < 0.01, *** denotes *p* < 0.001, and no asterisk indicates *p* > 0.05.

Pearson correlation analysis (Figure 9) showed that the density of zooplankton functional groups was correlated with most water environmental factors (Figure 9a). Specifically, significant correlations were observed with NH_4_^+^-N, SAL, NO_2_^−^-N, PO_4_^3−^-P, and NO_3_^−^-N (*p* < 0.05), while correlations with T, pH, and DO were weak (*p* > 0.05). Pearson correlation analysis revealed that only SAL (salinity) exhibited a significant negative correlation with the functional group RC (*p* < 0.01). Other environmental factors showed positive correlations with functional groups. Specifically, PO_4_^3−^-P had significant correlations with LCF, MCF, and SCC (*p* < 0.01); PO_4_^3−^-P showed significant correlations with the functional group PP (*p* < 0.05); RC had a significantly positive correlation with NO_2_^−^-N at the *p* < 0.01 level; and NH_4_^+^-N exhibited significant correlations with the functional groups PF, SCC, and LCF (*p* < 0.05). As shown in Figure 9b, the relationships among zooplankton functional groups were more complex. LCF showed significant positive correlations with MCC, SCC, MCF, and PF (*p* < 0.01). Pearson correlation analysis showed that SCF and RF exhibited significant differences (*p* < 0.01); MCC and SCC showed significant differences (*p* < 0.05); RF and SCF had significant differences (*p* < 0.01); and SCC and PF showed significant differences (*p* < 0.01). To demonstrate the importance of environmental factors and zooplankton functional groups (Figure 10). The network on the right utilizes Mantel tests to analyze the correlation between each environmental factor and the species. The width of the lines represents the absolute value of the correlation (Mantel *r*), the color of the lines indicates the range of Significance *p*-values (Mantel *p*), and the type of line (solid or dashed) denotes the positive or negative sign of the correlation coefficient.

**Figure 11:**
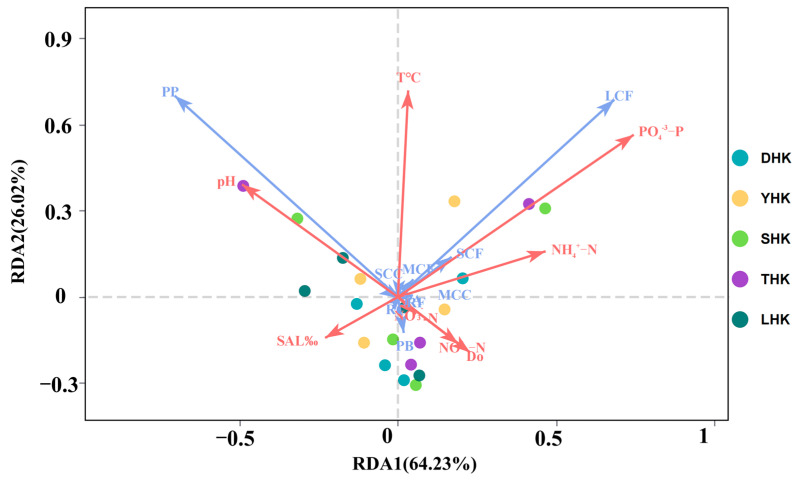
RDA of zooplankton functional groups and environmental factors.

The interpretation rate of RDA1 was 64.23%, that of RDA2 was 26.02%, and the cumulative interpretation rate of RDA1 and RDA2 was 90.25%. The results of the RDA (Figure 11) showed that the distribution of each sampling point was relatively scattered without an aggregation trend. On the RDA1 axis, pH and SAL showed negative correlations, while other environmental factors all showed positive correlations. On the RDA2 axis, T, NH_4_^+^-N, PO_4_^3−^-P, and pH showed positive correlations, while other environmental factors all showed negative correlations. The density of zooplankton functional groups is mainly influenced by factors such as NH_4_^+^-N, PO_4_^3−^-P, NO_2_^−^-N.

### 3.5. Study on the Ecological Niche and Model Prediction of Zooplankton Functional Groups

**Figure 12:**
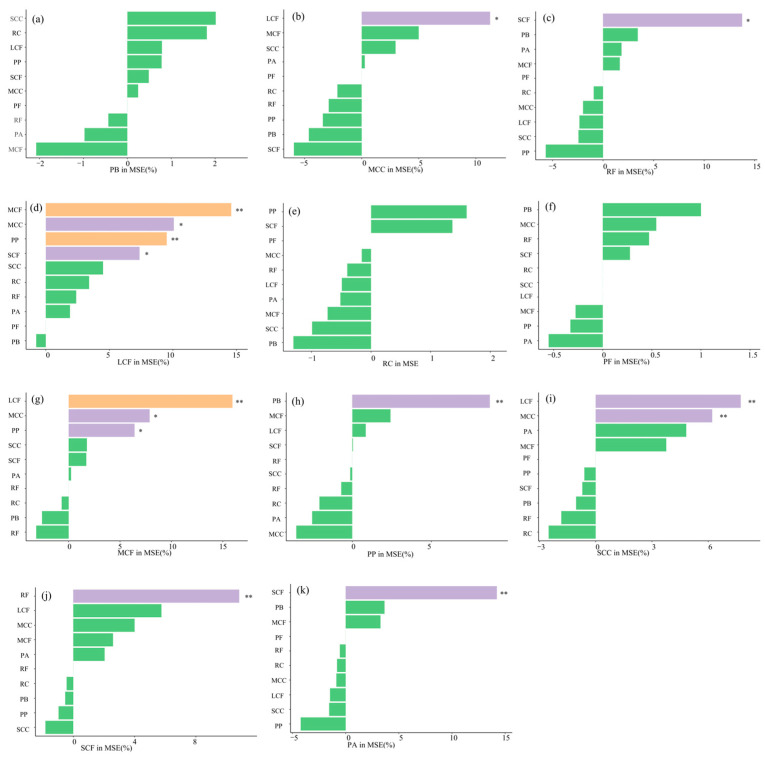
Prediction of the importance of mutual influence among functional groups of plankton in five estuaries of Qinhuangdao based on random forest model. Note: “*” *p* < 0.05, “**” *p* < 0.01. Note: (**a**) The significance of functional groups SCC, RC, LCF, PP, SCF, MCC, PF, RF, PA and MCF to the PB functional group; (**b**) The significance of functional groups SCC, RC, LCF, PP, SCF, PB, PF, RF, PA and MCF to the MCC functional group; (**c**) The significance of functional groups SCC, RC, LCF, PP, SCF, PB, MCC, RF, PA and MCF to the RF functional group; (**d**) The significance of functional groups SCC, RC, MCC, PP, SCF, PB, PF, RF, PA and MCF to the LCF functional group; (**e**) The significance of functional groups SCC, MCC, LCF, PP, SCF, PB, PF, RF, PA and MCF to the RC functional group; (**f**) The significance of functional groups SCC, RC, LCF, PP, SCF, PB, MCC, RF, PA and MCF to the PF functional group; (**g**) The significance of functional groups SCC, RC, LCF, PP, SCF, PB, PF, RF, PA and MCC to the MCF functional group; (**h**) The significance of functional groups SCC, RC, LCF, MCC, SCF, PB, PF, RF, PA and MCF to the PP functional group; (**i**) The significance of functional groups PP, RC, LCF, MCC, SCF, PB, PF, RF, PA and MCF to the SCC functional group; (**j**) The significance of functional groups PP, RC, LCF, MCC, SCC, PB, PF, RF, PA and MCF to the SCF functional group; (**k**) The significance of functional groups PP, RC, LCF, MCC, SCC, PB, PF, RF, SCF and MCF to the PA functional group.

Random forest model analysis showed that the strongest influencing factor for MCF was LCF, followed by MCC and PP, while RC, PB, and RF showed negative correlations. The main influencing factor for RA was SCF, followed by PB and MCF, with other factors showing negative correlations; PB showed negative correlations with RF, PA, and MCF but showed positive correlations with all others; PF showed negative correlations with MCF, PP, and PA but showed positive correlations with all others. The main influencing factor for PP was PB, followed by MCF and LCF, with other factors showing negative correlations; RF and SCF showed a significant positive correlation. Random forest model analysis showed (Figure 12) that LCF had extremely significant differences with MCF and PP (*p* < 0.01), showed significant positive correlations with MCC and SCF (*p* < 0.05), and only showed a negative correlation with PB; RC only had positive correlations with PP and SCF, with negative correlations to all other factors; SCC showed extremely significant positive correlations with LCF and MCC (*p* < 0.01), followed by PA and SCF, and negative correlations to all others; MCC showed a significant positive correlation with LCF (*p* < 0.05); and SCF showed an extremely significant positive correlation with RF (*p* < 0.01).

#### Niche Index, Interspecific Association, and Niche Overlap

**Figure 13:**
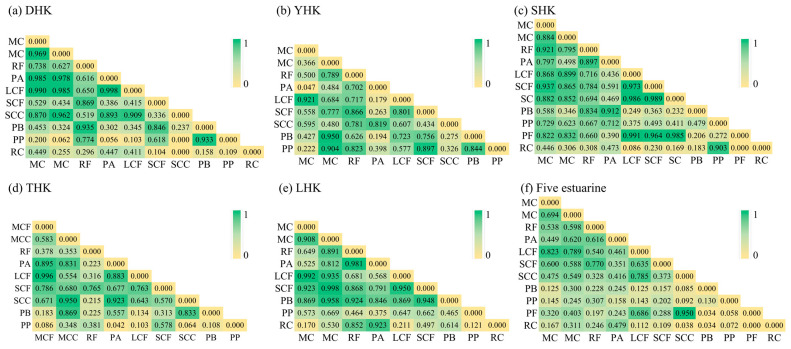
Ecological niche overlap index. (**a**) DHK, (**b**) YHK, (**c**) SHK, (**d**) THK, (**e**) LHK, (**f**) total value of five regions.

The niche overlap values of zooplankton functional groups in five estuaries along the Bohai nearshore during four seasons are shown in Figure 13. The niche overlap in the five estuaries ranges from 0.000 to 0.950. The niche overlap in DHK ranged from 0.000 to 0.998, with 16 pairs showing high overlap rates (Oik > 0.7). The highest overlap occurred between LCF and PA, while the lowest was between PP and SCC. In YHK, niche overlap ranged from 0.050 to 0.950, with 15 high-overlap pairs (Oik > 0.7). PB and MCC had the highest overlap, whereas PA and MCF showed the lowest. SHK exhibited niche overlap from 0.000 to 0.991, with 26 high-overlap pairs (Oik > 0.7). LCF and PF had the highest overlap, while PF and RC showed the lowest. THK’s niche overlap ranged from 0.042 to 0.996, with 11 high-overlap pairs (Oik > 0.7). The highest overlap was observed between LCF and MCF, while the lowest overlap was found between PP and SCC. LHK had niche overlap from 0.121 to 0.998, with 19 high-overlap pairs (Oik > 0.7).

**Figure 14:**
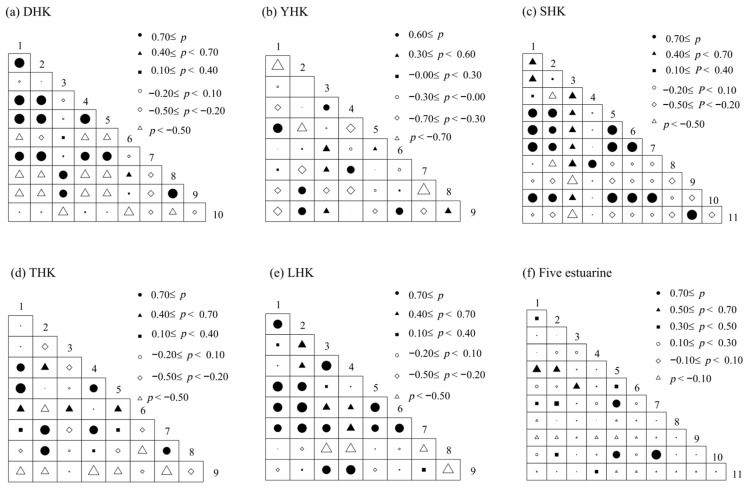
Pearson’s correlation analysis under different fertilization gradients. Note: (**a**) DHK, (**b**) YHK, (**c**) SHK, (**d**) THK, (**e**) LHK, (**f**) total value of five regions.

According to the Pearson correlation coefficient test results (Figure 14), DHK found 20 positive correlation pairs and 25 negative correlation pairs, including 13 pairs with significant positive correlations (correlation coefficient *r* > 0.7). YHK showed 18 positive and 18 negative correlation pairs, with 14 pairs showing significant positive correlations *r* > 0.7). SHK showed 17 positive and 38 negative correlation pairs, with only 3 pairs showing significant positive correlations (*r* > 0.7). THK showed 17 positive and 19 negative correlation pairs, including 7 pairs with significant positive correlations (*r* > 0.7). LHK showed 28 positive and 19 negative correlation pairs, with 7 pairs showing significant positive correlations (*r* > 0.7). Across all five estuaries, 31 positive correlation pairs and 24 negative correlation pairs were identified in the interrelationships of zooplankton functional groups, including 16 pairs with significant positive correlations (*r* > 0.7).

**Figure 15:**
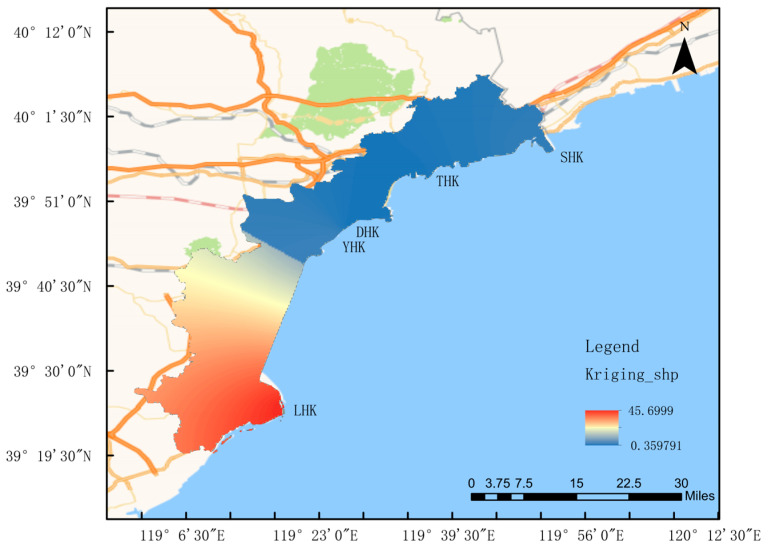
Association map of AC species in different regions.

The network diagram of interspecific relationships among zooplankton functional groups in different estuaries (Figure 15) revealed that DHK showed 3 pairs with positive correlations and 3 pairs with negative correlations. For YHK, 2 pairs showed positive correlations, and 1 pair showed negative correlation. For SHK, 1 pair showed a positive correlation, and 2 pairs showed significant negative correlations. For THK, 2 pairs showed significant positive correlations, 2 pairs showed positive correlations, and 6 pairs showed significant negative correlations. For LHK, 1 pair showed a positive correlation, and 1 pair showed a significant positive correlation. Across all five estuaries, the network diagram identified 21 pairs with positive correlations and 7 pairs with negative correlations.

## 4. Discussion

### 4.1. Composition and Diversity Analysis of Zooplankton Communities in Five Estuaries of Qinhuangdao City

Zooplankton are a group of small aquatic animals that feed on phytoplankton, possess weak swimming abilities, and live a planktonic lifestyle. They serve as important prey for fish and other aquatic animals, playing a crucial role as a link in the food chain [36,37]. Zooplankton are highly sensitive to environmental changes and, thus, are regarded as excellent indicator organisms for assessing ecosystem status. In aquatic systems, variations in physical-chemical and biological parameters can induce corresponding adjustments in the community structure and abundance of plankton [38,39]. Understanding the population dynamics and structural characteristics of their population plays a vital role in assessing fishery productivity in estuaries.

**Figure 16:**
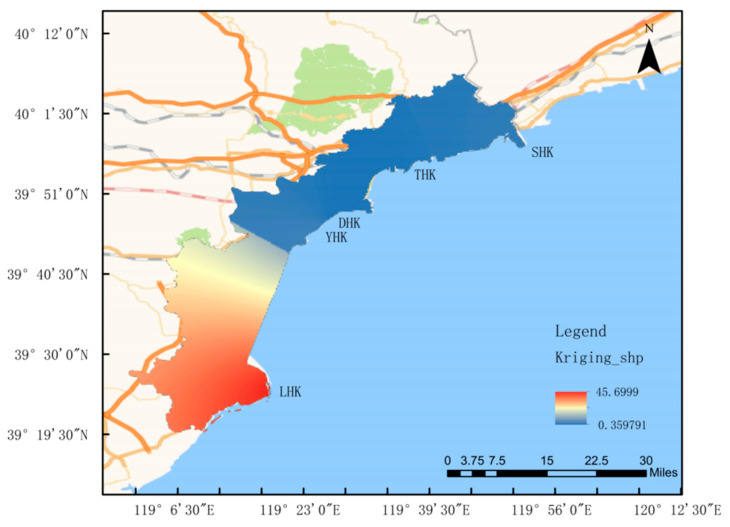
Distribution of flow rate in Qinhuangdao River, China.

The results of zooplankton surveys in four seasons at 15 sampling sites across 5 estuaries in the Qinhuangdao of the Bohai Sea showed that a total of 69 zooplankton species were collected. Fan Kai and Li Qingxue analyzed zooplankton samples from the Bohai Bay between 2003 and 2004. In four cruises, the first cruise (summer) had the highest species richness (30 species), followed by the fourth cruise (spring, 23 species), the second cruise (autumn, 19 species), and the third cruise (winter, 16 species). Species numbers showed seasonal variations, peaking in summer, being relatively high in spring, and decreasing in autumn and winter. Many species and larvae only appeared in summer or spring cruises, such as the appendicularian *Oikopleura dioica* (Urochordata), and various larvae, including *krill larvae*, zoea larvae of Brachyura, *megalopa* larvae, *Porcellanidae* larvae, *Stomatopoda* larvae, *holothurian* larvae, and fish larvae, indicating that most marine animals reproduce in spring and summer. These results are similar to those of this study, where summer had the highest species richness and there were fewer species in autumn and winter, with abundant larvae (*nauplii*) appearing in summer. In the autumn of 2013, zooplankton samples from the central Bohai Sea were identified, yielding 42 zooplankton species and 14 groups of planktonic larvae [40]. The species composition was similar to that of this study, dominated by Copepoda. Sha Jingjing investigated Bohai plankton during the summers of 2016–2018, finding that Copepoda also constituted the main component at 37% [41]. The high salinity in the estuaries of this study did not support the abundance of Rotifera species, consistent with previous Bohai zooplankton surveys. Many studies have shown that crustaceans dominate the species composition of zooplankton in estuarine areas, with Copepoda being the dominant group, as reported in [42] studies, which is consistent with the survey results of this study. The difference analysis in this study revealed that the average runoff of the LHK was significantly higher than that of other estuaries (Figure 16). Water quality index analysis indicated that salinity suggested freshwater would be stored for a certain period under the scouring of high runoff. Due to this factor, the Luanhe Estuary had far more unique species than the other four estuaries, including freshwater and brackish water species. The intense mixing of different water masses created a more complex water environment, which could support a greater diversity of organisms. Thus, the variation range of zooplankton groups was further expanded [43,44]. Compared to the species composition of historical data, the number of adult zooplankton species in this study has increased substantially, while the number of planktonic larval species has shown little difference, and *Copepoda* still dominates in species composition. Owing to the particularity of the estuary, numerous freshwater species have been added, resulting in a significant increase in the types of zooplankton compared with previous studies. This study focuses on the major estuaries of the Bohai Sea, where abundant nutrients and unique hydrology lead to differences in species diversity and growth conditions [45].

In the surveyed sea area, THK exhibited high abundance and biomass but low biodiversity, as the high abundance was concentrated in a few species, such as *Noctiluca* and *Calanus sinicus*. He Yutao et al. [46] pointed out that the surge in the number of dominant species would reduce the biodiversity of zooplankton. The results of this study showed that *Cladocera* were the most dominant species in DHK and YHK, with *Calanus sinicus* and immature *nauplii* as the most dominant species, which collectively represented nearly the entire LCF and SCF. *Cladocera*, with their relatively large body size, play significant roles in aquatic ecosystems. On the one hand, they can directly affect the abundance of phytoplankton in water bodies, while on the other hand, they serve as natural prey for fish and other aquatic organisms [47]. According to relevant literature analysis, seawater temperature, salinity, phytoplankton abundance, DO, pH, and water masses are key factors affecting the spatiotemporal distribution characteristics of zooplankton [48,49]. This study reveals that salinity and nutrients are the primary drivers of significant differences in species composition among the five estuaries. In SHK, THK, and LHK, *Noctiluca* (Dinophyta) was the dominant species in March, September, and December, in addition to Cladocera. Zhou Zunchun et al. [50] demonstrated that low salinity is more conducive to the reproduction of *Noctiluca*. This study found a negative correlation between the abundance of *Noctiluca* and salinity. The salinity in LHK and THK was lower compared to the other five estuaries.

Additionally, diatoms, the main prey of *Noctiluca*, were dominant in the phytoplankton communities of the coastal Bohai Sea and estuaries (with a relative abundance of up to 57%) [51,52], both of which facilitated the massive reproduction of *Noctiluca* in estuaries. The shallow depth and rich nutrients in estuarine areas may also be important reasons for the massive aggregation and reproduction of *Noctiluca* in these areas. Additionally, in THK, *Noctiluca* accounted for over 90% of the species in September, possibly because human activities have a more intense influence on the coastal areas of THK. The discharge of domestic sewage has increased the contents of nitrite nitrogen (NO_2_^−^-N) and nitrate nitrogen (NO_3_^−^-N), which further promotes the formation of algal blooms. As proposed in Wang Songbo’s paper Effects of Light and Nutrients on Biomass and Trophic Linkages of Zooplankton and Phytoplankton [53], an increase in nutrients exacerbates chlorophyll concentration, and higher surface chlorophyll concentration can provide abundant prey for *Noctiluca*. Changes in the relative and absolute densities of zooplankton communities can affect the productivity of higher trophic levels by altering secondary production rates and energy transfer efficiency. *Noctiluca* can form competitive relationships with zooplankton by preying on phytoplankton and directly feeding on zooplankton eggs or larvae, thereby influencing the community structure of zooplankton [54]. The discussion has certain limitations due to the lack of specific chlorophyll a indices.

### 4.2. Changes in Functional Community Characteristics of Zooplankton

The density and biomass of zooplankton vary across different habitats, and their composition also differs [55,56]. Therefore, the spatiotemporal distribution of estuarine zooplankton exhibits more pronounced variations than other water bodies, given the complex and dynamic ecological environment in estuarine areas, where hydrodynamic conditions and physical-chemical properties undergo drastic spatiotemporal changes. The significant fluctuations in water levels caused by variations in runoff in each estuary, coupled with anthropogenic disturbance factors in adjacent coastal areas, are important reasons for the changes in species composition. This also constitutes the main reason why the community structure of THK differs substantially from that of other estuaries. This result is consistent with the findings of Gutiérrez et al. [57], who surveyed zooplankton in the humid regions of the lower Paraná River Delta during an extreme drought event. Their results indicated that zooplankton exhibited a significant increase in taxonomic diversity and substantial fluctuations in biomass during the low-water period.

Bray–Curtis analysis showed that the zooplankton functional groups in the five Bohai Sea estuaries exhibited significant differences across different estuaries and seasons. The zooplankton community composition throughout the basin was non-uniform, with spatiotemporal variations in species richness and biomass [58]. The survey showed that with the increase in temperature and light intensity in June, the growth rate of zooplankton accelerated, dormant eggs floated to the water surface for hatching, and the number of species increased rapidly, promoting the improvement of diversity [59]. The LCF of zooplankton almost completely dominated in June. The zooplankton functional groups transitioned from being dominated by *nauplius* of the SCF in April to being dominated by *Calanus sinicus* of the large zooplankton LCF in June. By September, DHK and YHK gradually shifted back to being dominated by *copepod nauplii* and other *copepods*. The massive bloom of *Noctiluca* in the PP functional group in THK may be directly related to temperature and nutrient availability [60]. *Noctiluca* can form competitive relationships by preying on phytoplankton and zooplankton and directly feeding on zooplankton eggs or larvae, leading to a significant reduction in other species. In December, the dominant functional groups in THK also showed significant differences from those in other estuaries, which were mainly dominated by the PB functional group (bacteria-feeding) [24]. This may be related to the competition formed between the massive bloom of *Noctiluca* in September and most *rotifers* and *copepod* larvae, resulting in the gradual dominance of the PB functional group that feeds primarily on bacteria [51]. The feeding habits of dominant *copepod* in estuarine zooplankton often undergo seasonal changes. In the summer, phytoplankton is the most important carbon source for zooplankton, while other carbon sources are utilized in other seasons [61]. This finding is similar to the results reported by Bu Yaqian [62] in the paper titled “Community Structure of Zooplankton and Its Relationship with Environmental Factors in the Bohai Sea and North Yellow Sea during Summer and Winter”. Due to feeding size constraints, only three sources are likely to play a major role: organic detritus, microheterotrophs, and microphytobenthos [63]. The overall functional group shift in estuaries showed a transition from SCF to LCF. SCF, feeding on bacteria, algae, organic matter, and protozoa, occupies the most dominant position in aquatic ecosystems. The core function of the zooplankton functional group SCF is to regulate the cycling of nutrients in the water body and inhibit the growth of harmful algae through ecological processes such as feeding and metabolism, thereby maintaining the stability of the aquatic environment [64]. Therefore, it is inferred that the SCF functional group of zooplankton plays a crucial role in regulating estuarine aquatic ecosystems [65].

### 4.3. Interaction of Zooplankton Functional Groups and Their Relationship with Water Environmental Factors

In recent years, the study of zooplankton interspecific interactions has gradually been carried out; however, the study of zooplankton interactions in estuarine areas remains relatively underexplored [66]. Generalist species exhibit strong adaptability to their environment, characterized by a broad niche breadth. As their population density increases, interspecific competition intensifies accordingly. Competition and predation among zooplankton are also important factors influencing population dynamics [67]. The functional groups in different niches may have predatory relationships, which cause predators and prey to influence each other, thereby jointly affecting the dynamic changes in community structure [68]. Temperature, nutrients, bottom-up effects of phytoplankton, top-down effects of fish predation, interspecific competition, and other factors are the primary factors influencing the growth of zooplankton, which also affect the distribution of zooplankton functional groups [69].

Plankton plays a key role in regulating the balance of aquatic ecosystems, and temperature is a key environmental factor affecting the growth of phytoplankton. The functional groups of zooplankton exhibit a significant response to changes in phytoplankton [27]. Evans L.E. and other scholars monitored zooplankton in the Atlantic Ocean over 57 years and explored the relationship between surface water temperature and changes in community size. They found that the mean size of copepod communities showed a negative correlation with temperature, and the community followed Bergmann’s rule [70]. Due to the uniqueness of the estuary, this study, using Redundancy Analysis (RDA), found that temperature was not the primary driving factor influencing the composition of zooplankton communities. Instead, factors such as phosphate, nitrite, and salinity (SAL) had a greater impact on the functional groups of zooplankton, with phosphate being the most significant driver. This result is consistent with the findings of Jiang Xiaojun [71] in the study “Response of Light and Zooplankton Community Structure to Nutrient Concentration Changes Induced by Hydrodynamic Characteristics”.

The competitive relationships among zooplankton have important implications for phosphorus regulation. In high-phosphorus environments, certain zooplankton populations exhibit stronger phosphorus utilization capabilities, enabling them to inhibit the growth of other species [72]. This competitive phenomenon implies that in resource-rich environments, certain zooplankton will occupy more phosphorus resources, leading to phosphorus scarcity for other species. As a result, the density distribution of zooplankton will change, and the structure of the entire ecosystem will also be affected. Such competitive relationships may arise from the specific mechanisms by which zooplankton adapt to high-phosphorus environments [72]. The PO_4_^3−^-P concentration in June of this study was significantly higher than that in other months, and the density and biomass of zooplankton functional groups were also much higher than those in other months. Correlation analysis in this study showed a significant positive correlation between PO_4_^3−^-P and functional group density. Salinity changes can also increase phytoplankton diversity, and species tolerant to high concentrations of nutrients, such as algae, become dominant. Energy flow in the food chain makes salinity changes unfavorable for the development and density increase in predators in medium-to-large SCF and LCF communities [73]. Correlation analysis revealed a significant negative correlation between salinity and all zooplankton functional groups except for PB. The PB functional group is small in size (mostly microprotozoa), with a specialized ecological niche and low competitive pressure. High-salinity environments filter out most salt-intolerant zooplankton groups, reducing predation pressure and spatial competition on the PB functional group, thereby enabling it to focus on utilizing halotolerant bacterial resources for population maintenance. In contrast, other functional groups are mostly located in the middle of complex food chains and are more strongly affected by the cascading effects of high salinity on upper and lower trophic levels, making it difficult for them to achieve high-salinity adaptation independently [74].

Pearson correlation analysis revealed that interspecific competition was also a significant factor influencing the functional groups of zooplankton. In this study, zooplankton were further divided into functional groups. Except for PP, PB, and PA, which showed no significant correlation with other functional communities, the functional groups LCF, SCF, MCC, MCF, RF, SCC, and RF all exhibited significant correlations with other functional communities. DHK and YHK are both dominated by the SCF and LCF. The functional groups LCF, SCF, and MCF primarily feed on bacteria and algae. Fluctuations in these food sources will further affect the abundance of filter-feeding zooplankton populations and indirectly impact predatory zooplankton [24]. Studies have shown that the body size of zooplankton exhibits a negative correlation with food resources, and fish predation has also led to a decrease in the body size of zooplankton [75].

Estuaries, as habitats for the early-life stages of fish, may see a decrease in zooplankton density related to the growth and development of these early fish resources. This is consistent with the study by De Meester et al., which proposed that the composition of fish populations directly affects the composition of zooplankton communities [76]. THK’s inshore area has factories, resulting in significant human disturbance. The nutrients and water environment are more suitable for the growth and development of *Noctiluca*, leading to a full-scale bloom in THK in September. The bloom of *Noctiluca* affected the composition of the entire zooplankton community structure such that by December, the PB functional group had become the dominant functional group. When *Noctiluca* blooms occur, the abundance of *Calanus sinicus* decreases. This reduces the grazing pressure on phytoplankton such as the family Chattonellaceae, leading to an increase in the abundance of these phytoplankton species. Furthermore, the decomposition of phytoplankton detritus exerts an impact on bacterial communities [77]. The residual effects of algal bloom recession require specific ecological conditions to be triggered. When *Noctiluca* dies and decomposes, it consumes dissolved oxygen in the water and releases nutrients. These nutrients may accumulate in the water column until December, when favorable conditions such as water temperature and salinity promote the massive reproduction of bacteria. This in turn provides a survival basis for the PB functional group and bacteriophages, which is consistent with the water quality conditions reported in this study [78]. This may be one of the reasons why the dominant species in THK differ from those in the other four estuaries. To further explore the interaction relationships among species, this study conducted cluster analysis on zooplankton. The results showed that the higher the similarity between species, the more significant their negative correlation. Due to their high similarity in living habits and feeding preferences, these species often occupy the same ecological niche, leading to increased predation pressure and thus exhibiting significant negative correlations in their interactions [79]. Conversely, species from different functional groups exhibit lower similarity, occupy distinct ecological niches, and primarily act as prey or engage in cooperative relationships, thus demonstrating primarily positive correlations in their interactions [80]. The growth of zooplankton is jointly influenced by factors such as water flow velocity, water quality nutritional status, salinity (SAL‰), PO_4_^3−^-P, phytoplankton, and predator pressure [81]. The distribution and dynamic changes in zooplankton functional groups in the coastal estuarine areas of the Bohai Sea are primarily controlled by the interactions among functional groups and water environmental factors, with the former having a more significant impact.

### 4.4. Quantitative Evaluation of Functional Group Ecological Niche Contribution Model

This study used a random forest model to predict the importance indices of interactions among zooplankton functional groups. The main positive factors influencing interspecific relationships of zooplankton functional groups in the coastal estuaries of the Bohai Sea were MCF, MCC, PP, SCF, LCF, and RF. According to the model prediction, an increase in the RF leads to a rapid increase in large and medium-sized zooplankton such as LCF, MCC, SCF, and MCF. MCC, MCF, and LCF feed on bacteria, fungi, and small *rotifers*. With the increase in RF, the food supply becomes abundant, reducing competition among these functional groups and leading to a simultaneous increase in their numbers [69]. When the LCF population reaches a certain density, it engages in interspecific competition with SCF and MCC for shared food resources, leading to the suppressed abundance of SCF and MCC due to this resource competition. RF may be preyed upon by MCF, and its abundance decreases accordingly when MCF increases.

As a special functional group, PP exhibits different roles in various stages of the ecosystem. When copepod zooplankton are in the larval stage, the PP functional group not only competes with zooplankton larvae but also directly preys on them, thereby inhibiting the abundance of zooplankton larvae. This is consistent with the model’s prediction of a negative correlation between the SCF functional group and PP, and this predictive result aligns with recent studies on the interactions among zooplankton functional groups [81,82,83]. To further explore the interrelationships of zooplankton functional groups, an in-depth analysis was conducted on their niche overlap and interspecific relationships. From the perspective of survival strategies, the interactions among zooplankton are extremely intricate, where most relationships in large environments manifest as synergistic interactions that mutually promote co-resistance to external environmental pressures. In small environments with limited space, species primarily engage in mutual predation and competition [84]. The results of the correlation network diagram of AC in this study are consistent. Studies have shown that as the environment changes, the competitiveness of species in resource utilization also changes. When resources are abundant, species do not need to compete for shared resources and can fully occupy their respective adaptive resource dimensions and living spaces, achieving niche differentiation and thereby leading to a decrease in niche overlap. This is consistent with the logic in classical ecology that “when resources are rich, competition eases, and species can achieve stable coexistence through niche expansion.” Conversely, when resources are scarce, species engage in direct competition for limited resources. In the short term, due to the failure to complete adaptive niche differentiation, they still need to share key resources, which is manifested as an increase in niche overlap. This phenomenon is not contradictory to traditional theories such as the competitive exclusion principle—traditional theories emphasize that on a long-term evolutionary scale, intensified competition will drive species to achieve niche separation by adjusting resource utilization strategies, ultimately reducing overlap to avoid elimination [85]. The competition for nutrient resources among species with high niche overlap exhibits significant asymmetric characteristics [86]. Species with broader niches, due to their high degree of resource utilization and wide distribution, are more likely to exhibit a high degree of niche overlap with other species. When the homogenizing selective pressure exerted by fish top-down control and zooplankton trophic cascade effects is applied to the ecosystem, substitutive competitive responses will arise among functionally redundant species. Specifically, biomass fluctuations of a single species can trigger trophic cascade effects, which, through the reconstruction of resource allocation patterns, lead to dynamic responses across multiple trophic levels. This dynamic balancing process is constrained by a two-way regulatory mechanism of interspecific competitive exclusion and coevolution, ultimately forming non-linear community succession outcomes, which are consistent with the research conclusions of Ju Yongfu et al. [87] and Elmhagen B et al. Through model quantification and niche assessment, the mutually restrictive and collaborative relationships among zooplankton functional groups have been clarified, further exploring the ecological relationships among zooplankton. This lays a foundation for maintaining the diversity and population stability of zooplankton in the Bohai Sea and even semi-enclosed marine areas. As typical semi-enclosed marine ecosystems, such sea areas serve as spawning grounds, nursery grounds, and feeding grounds for numerous marine organisms. Meanwhile, the structure of their zooplankton communities is significantly influenced by environmental factors such as temperature and salinity, as well as human activities. Maintaining the population stability and diversity of zooplankton in these areas is of great significance for the material cycling, energy flow of regional ecosystems, and the sustainability of fishery resources.

## 5. Conclusions

The monitoring results of zooplankton in the DHK, YHK, SHK, THK, and LHK estuaries of the Qinhuangdao coastal area in the Bohai Sea across four seasons of 2024 showed that the diversity index and richness index of zooplankton in summer were significantly higher than those in winter (*p* < 0.05).Based on body size, feeding habits, reproductive type, life cycle, and escape ability, a classification system containing 17 zooplankton functional groups was established, and a total of 11 functional groups were detected in the monitoring area of this study.The dominant functional groups were consistent across all seasons in four estuaries (DHK, YHK, SHK, and LHK), with both SCF and LCF serving as the dominant groups. In contrast, the dominant functional groups in THK were PP and PB, which showed a distinct difference from the other estuaries.Significant spatiotemporal variations were observed in water temperature (T), phosphate (PO_4_^3−^-P), nitrite (NO_2_^-^-N), and salinity (SAL) across the five estuaries. However, the seasonal variation trends of pH and dissolved oxygen (DO) overlapped significantly, with no remarkable spatiotemporal differences.Results from niche overlap analysis and AC interspecific association analysis indicated that there were significant differences in the interspecific relationships of zooplankton across different spatial scales:At the small-scale level, the zooplankton populations were dominated by competition and predation, showing negative correlation relationships;At the large-scale level, the zooplankton functional groups were mainly characterized by mutually promoting synergistic relationships.Among these groups, SCF and LCF continued to play a dominant role in the community, and the interspecific relationships were more critical in shaping the zooplankton community structure.

## Figures and Tables

**Table 1:** Descriptor zooplankton functional groups in freshwater ecosystem.

Functional Group	Size/mm	Feeding Habit
Rotifers filter feeders, RF *	-	Feed on bacteria, algae and organic matter
Rotifers carnivora, RC *	-	It feeds on protozoa, other rotifers and small crustaceans
Rotifers predators, RP	-	It feeds mainly on algae
Small copepods and claocera filter feeders, SCF *	<0.7	Feed on bacteria, algae, organic matter and protozoa
Small copepods and claocera carnivora, SCC *	<0.7	They feed on rotifers, brachiopods, dipteran insects (larvae of midges) and oligochaetes
Middle copepods and claocera filter feeders, MCF *	0.7~1.5	Feed on bacteria, algae, organic matter and protozoa
Middle copepods and claocera carnivora, MCC *	0.7~1.5	They feed on rotifers, brachiopods, dipteran insects (larvae of midges) and oligochaetes
Large copepods and claocera filter feeders, LCF *	>1.5	Feed on bacteria, algae, organic matter and protozoa
Large copepods and claocera carnivora, LCC	>1.5	They feed on rotifers, brachiopods, dipteran insects (larvae of midges) and oligochaetes
Protozoan photosynthetic autotrophs, PP *	-	Flagellates with chromophores capable of photosynthesis and groups with symbiotic green algae
Protozoan algivores, PA *	-	They feed on algae
Protozoan bacterivores, PB *	-	It feeds on bacteria
Protozoan detritivores, PD	-	Feeds on organic detritus and bacteria
Protozoan fungivores, PF *	-	They feed on bits of food
Protozoan saprotrophs, PS	-	It feeds on large organic molecules dissolved in water
Protozoan raptors, PR	-	It feeds on protozoa such as flagellates, trichurans and ciliates or small rotifers
Protozoan nonselective omnivores, PN	-	It eats both bacteria and debris, as well as algae

Note: The functional groups assigned in this study are marked with *. Functional groups marked with ‘-’ are classified based on ‘feeding habit + reproductive type,’ and body size is not used as a classification indicator.

## Data Availability

The data supporting this study’s findings are available from the corresponding authors upon reasonable request.
